# Comparative impact of two continuing education activities targeted at COPD educators on educational outcomes: protocol for a non-randomized controlled study using mixed methods

**DOI:** 10.1186/s12913-018-3284-6

**Published:** 2018-06-18

**Authors:** Myriam Gagné, Jocelyne Moisan, Sophie Lauzier, Christine Hamel, Patricia Côté, Jean Bourbeau, Louis-Philippe Boulet

**Affiliations:** 1 Knowledge Translation, Education and Prevention Chair in Respiratory and Cardiovascular Health, Laval University, Quebec City, QC Canada; 2 Quebec Heart and Lung Institute, Laval University, Quebec City, QC Canada; 3 Population Health and Optimal Health Practices Research Unit, CHU de Quebec Research Center, Quebec City, QC Canada; 4 Faculty of Pharmacy, Laval University, Quebec City, QC Canada; 5 Faculty of Education, Laval University, Quebec City, QC Canada; 6 Quebec Respiratory Health Education Network, Quebec City, QC Canada; 7 Respiratory Epidemiology and Clinical Research Unit, Research Institute of the McGill University Health Center, McGill University, Montreal, QC Canada; 8 Faculty of Medicine, McGill University, Montreal, QC Canada; 9 Faculty of Medicine, Laval University, Quebec City, QC Canada

**Keywords:** Education, continuing, Pulmonary disease, chronic obstructive, Patient education as topic, Revised Bloom’s taxonomy

## Abstract

**Background:**

Therapeutic patient education (TPE) improves quality of life and reduces health care utilization among patients with chronic obstructive pulmonary disease (COPD). However, benefits from TPE might depend on the performance of the educators and training is needed to ensure the effective delivery of TPE interventions. Based on the framework by Moore et al. (J Contin Educ Health Prof 29:1-15, 2009), we will compare the impact of two continuing education (CE) activities on TPE in regard to the following educational outcomes: (1) learning, (2) self-report of competence, (3) performance of the educators, and (4) outcomes of COPD patients who will meet the newly trained educators for TPE.

**Methods:**

We will conduct a non-randomized controlled study using mixed methods. Educators will first participate in a CE activity on TPE that will include a role-playing simulation (experimental group) or in a lecture on TPE (comparison group) and then will perform TPE in COPD patients. Among educators, we will assess: (1) learning, by measuring knowledge about TPE, and (2) self-report of competence using self-administered questionnaires before and after the activity. Then, after the CE activity, we will assess (3) educators’ performance levels in delivering TPE by rating a videotaped TPE intervention. In COPD patients who will meet the newly trained educators for TPE after either CE activity, we will assess (4) quality of life and resource utilization using interviewer-administered questionnaires, before and after TPE. Statistical analyses will compare the experimental group against the comparison group using multivariate models. Using a semi-structured interview guide, we will conduct interviews with educators and perform content analysis. Results will be integrated in order that qualitative results further explain the quantitative ones.

**Discussion:**

To the best of our knowledge, this is the first controlled mixed methods study to compare the impact of two CE activities on TPE in regard to four educational outcomes. We believe this study will serve as a model for evaluating CE activities on TPE. Results from this study could increase educators’ performance levels in delivering effective TPE interventions, and, in turn, COPD patient outcomes.

**Trial registration:**

The study was registered on https://clinicaltrials.gov/ (NCT02870998) on March 15, 2016.

**Electronic supplementary material:**

The online version of this article (10.1186/s12913-018-3284-6) contains supplementary material, which is available to authorized users.

## Background

Chronic obstructive pulmonary disease (COPD) is an irreversible, but treatable disease that is characterized by airflow limitation and persistent respiratory symptoms [1]. The global prevalence of COPD is estimated at 12% (95% confidence interval, or CI, 8–15%) among individuals aged 30 years or over [2].

The global burden of COPD is considered to be high [3]. In particular, results from a systematic review indicate that COPD is responsible for a large number of emergency room visits and hospitalizations, profoundly impacts patients’ quality of life, and results in expensive total costs per COPD patient from both the patient and societal perspective [4].

To reduce COPD-related morbidity, COPD patients need to develop several self-management skills, including: using their inhalers correctly, monitoring the control of their disease, preventing and controlling exacerbations, and using breathing techniques [1].

Therapeutic patient education (TPE) is an active, evidence-based, and patient-centered process by which health care professionals, also referred to as educators, guide and support patients in developing these self-management skills [5]. When compared to usual care, TPE results in improvement of quality of life among COPD patients, as well as in reduction of dyspnoea and hospitalizations [6].

Nevertheless, not all TPE programs have a positive impact on COPD health outcomes. For instance, in 2012, a randomized controlled trial by Fan et al. [7] was terminated before its planned completion date, due to the excess mortality estimated within the participants allocated to TPE, compared to guideline-concordant usual care. Although a subsequent meta-analysis concluded that TPE does not result in an increased risk of mortality in patients with COPD [8], Fan et al. [7] were unable to explain why the number of deaths was higher in the TPE group than in the comparison group, nor were they able to assess the quality of TPE when reporting the results of their study. In this context, authors have hypothesized that benefits from TPE could depend on the performance of the educators and especially on the training that they received to deliver effective TPE interventions [9]. Hence, ensuring the optimal training of COPD educators is crucial.

Continuing education (CE) activities (including: lectures, conferences, seminars, workshops, symposia, and courses) have been widely used in order to improve health care professional practice and, subsequently, patient outcomes [10]. CE activities can be described through their learning objectives, activities, and assessments, and classified into six cognitive processes and four knowledge types according to the *Revised Bloom’s Taxonomy for Learning, Teaching, and Assessing* [11]. Conceptually, to promote translation of knowledge into practice, the learning objectives of a particular CE activity should be classified into the higher-order cognitive processes [11]. The correspondence between the learning objectives, activities, and assessments is defined as *alignment*. Described as *strong, weak*, or *misalignment*, alignment ensures the following two characteristics: (1) the learning activities enable attendees to perform adequately in assessments, and (2) the results of the assessments reflect the achievement of the learning objectives [11].

CE activities can also be described through some components of a CE activity that have been reported to impact on health care professional performance [12–14] and patients’ outcomes [13, 14]. These components include the following:

1. the number of attendees in a CE activity: small groups of < 10 attendees might result in better educational outcomes than moderate-to-large groups of ≥10 attendees [14];
2. the format of the CE activity: an active learning format that includes role play simulations, case discussions, or opportunities to practice skills might result in better educational outcomes than a passive learning format that includes: lectures or presentations, with or without question and answer periods [13, 14];
3. the organizational support [12].

Moore et al. [15] developed the *Expanded Outcomes Framework for Planning and Assessing Continuing Medical Education Activities* to evaluate the achievement of desired educational results. This framework includes several levels of assessment (Table 1). Based on this framework, our study will aim to compare the impact of two CE activities on TPE that target COPD educators. One CE activity will include a role-playing simulation and involve a small group of attendees (experimental group). The other CE activity will consist of a lecture on TPE that will be presented to a moderate-to-large group of attendees (comparison group).

**Table 1:** The expanded outcomes framework for planning and assessing continuing medical education activities by Moore et al. [15]: Definitions

Educational outcome	Definition
Satisfaction	“The degree to which the expectations of the participants about the setting and delivery of the [CE] activity were met.”
Learning	“The degree to which the participants state *what* the [CE] activity intended them to know [or] the degree to which participants state *how* to do what the [CE] activity intended them to know how to do [it].”
Competence	“The degree to which participants *show* in an educational setting *how* to do what the [CE] activity intended them to be able to do.”
Performance	“The degree to which participants *do* what the [CE] activity intended them to be able to do in their practices.”
Patient outcome	“The degree to which the health status of patients improves due to changes in the practice behavior of participants.”

Definitions are quoted from Moore et al. [15]

*CE* Continuing education

Specifically, we will compare both activities in regard to the following educational outcomes: (1) satisfaction, (2) learning, (3) self-report of competence, (4) performance of the COPD educators who will attend either CE activity, and (5) outcomes of COPD patients who will meet the newly trained COPD educators for TPE after the CE activity.

As reported in other studies assessing satisfaction with a CE activity [16–19], we hypothesize that (1) satisfaction with the CE activity will be similar in the experimental and comparison groups immediately after the CE activity. In view of the fact that a small number of attendees and an active learning format have been reported to improve health care professional performance and patient outcomes [13, 14], we hypothesize that: (2) improvements in educators’ learning before and after the CE activity will be greater in the experimental group (small group of educators + active learning format), compared to the comparison group (moderate-to-large group of educators + passive learning format), as will be (3) improvements in educators’ self-reports of competence before and after the CE activity, and (4) educators’ performance levels after the CE activity. Similarly, we believe that (5) improvements in patient outcomes before and after TPE will be greater in the experimental group (TPE delivered by educators who attended the experimental CE activity), compared to the comparison group (TPE delivered by educators who attended the comparison CE activity).

## Methods

The following study protocol adheres to the SPIRIT guidelines [20].

### Study design

We will conduct a pragmatic non-randomized controlled study using explanatory sequential mixed methods [21]. From June 2016 to October 2017, moderate-to-large groups of ≥10 educators will participate in a lecture on TPE (comparison group). From November 2017, and thereafter, small groups of < 10 educators will be involved in a CE activity on TPE that will include a role-play simulation (experimental group). In educators, quantitative and qualitative measurements will be undertaken before and after the CE activity. Four months after the CE activity, educators will provide COPD patients with TPE in their work settings. Measurements will be undertaken in COPD patients before and after TPE.

Our study was registered in ClinicalTrials.gov (NCT02870998) on March 15, 2016.

### Eligibility criteria

All French-speaking educators (e.g. nurses, respiratory therapists, and other allied health professionals) who will attend either CE activity on TPE organized on behalf of the *Quebec Respiratory Health Education Network* in June 2016, and thereafter, will be eligible to participate in the study. Most of these educators work in family medicine groups, local community service centers, or in hospitals, which include emergency departments. Physician-diagnosed COPD patients, who will meet the newly trained COPD educators for TPE after either CE activity, will be eligible to participate in the study.

### Interventions

Educators will be allocated to a CE activity on TPE that will include a role-playing simulation and involve a small group of attendees (experimental group) or to a lecture on TPE that will be presented to a moderate-to-large group of attendees (comparison group). Hence, the CE activities differ in their number of participants and learning format.

Both CE activities will be similar in regard to their content and learning objectives. The content of the CE activities will pertain to TPE and the general learning objective of both CE activities can be described as: to be able to deliver effective TPE interventions to COPD patients. The specific learning objectives are adapted from the Canadian Network for Respiratory Care’s *National Certified Respiratory Educator Learning Objectives* [22]. They are presented in Table 2.

**Table 2:** Specific objectives of the two CE activities

1.	To define TPE	
2	To demonstrate TPE skills while:	
	a.	Demonstrating how to take a patient’s medical history
	b.	Demonstrating how to teach a patient to use an action plan
	c.	Demonstrating how to teach a patient to complete a symptom diary form
	d.	Interpreting a patient symptom diary as to whether COPD control is acceptable
	e.	Demonstrating how to teach a patient to use and maintain medication delivery devices
	f.	Demonstrating pursued lip breathing techniques, diaphragmatic breathing, controlled cough and forced expiration techniques, and relaxation techniques
	g.	Performing a follow-up visit

These specific objectives are based on the Canadian Network for Respiratory Care’s *National Certified Respiratory Educator Learning Objectives* [22]

*CE* Continuing education, *COPD* Chronic obstructive pulmonary disease, *TPE* Therapeutic patient education

The study coordinator (M.G.) will attend the CE activities to ensure adherence to intervention protocols.

#### Experimental group: small groups of < 10 educators attending a CE activity on TPE that will include a role-playing simulation

In the experimental group, small groups of ≈6 educators will participate in a seven-hour CE activity on TPE that will include a role-playing simulation. As part of the development phase of this activity, the learning activities and assessments were reviewed by experienced case managers (*n* = 4) and physicians (*n* = 2). On four occasions, the activity was pretested among both experienced and non-experienced educators (n ≈ 4 per occasion).

Before the activity, educators will be asked to read a necessary online document on TPE. Learning activities will include the following:

- a lecture on core concepts of TPE (1.5 h; passive learning format);
- a group discussion about TPE (0.5 h; active learning format);
- demonstrations and case studies on how to demonstrate TPE skills while taking a COPD patient’s medical history and interpreting an action plan (two hours; passive and active learning format);
- demonstrations and practices of how to ensure TPE skills while teaching a patient to use and maintain medication delivery devices (one hour; passive and active learning format);
- a role-playing simulation relating to a COPD patient’s initial and follow-up TPE visits (two hours; active learning format).

**Fig. 1:**
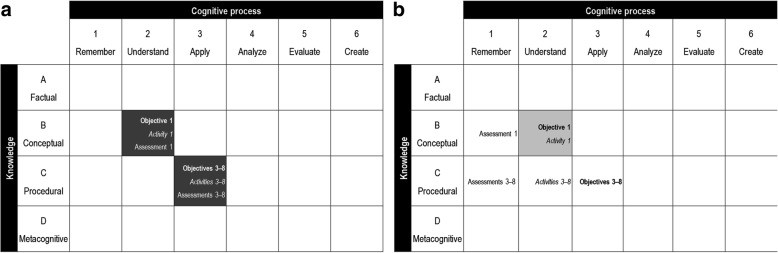
Alignment of the objectives, learning, and evaluation activities in the experimental and comparison groups. In accordance with the *Revised Bloom’s Taxonomy* [11], dark shading indicates that there is a strong alignment between the objectives, learning activities, and assessment (all present in the same cell), as in the experimental group (**a**). Light shading indicates that there only is a weak alignment. When the objectives, activities, and assessments are not in the same cell, there is a misalignment, as in the comparison group (**b**)

As part of the evaluation of the CE activity, participants will be asked the following two requests: (1) to fill a questionnaire assessing their comprehension of the core concepts of TPE, and (2) to perform an objective structured clinical examination with a standardized patient. Hence, the learning objectives, activities, and assessments will be strongly aligned, according to the *Revised Bloom’s Taxonomy* [11] (see Fig. 1a).

#### Comparison group: moderate-to-large group of ≥10 educators attending a lecture on TPE

In the comparison group, moderate or large groups of ≈12–25 educators will participate in a seven-hour lecture on TPE. Although the animator will encourage educators’ active participation during the presentation, learning will be mostly passive, because a lecture format does not easily allow for an attendee-animator dialogue [23]. The evaluation of this CE activity will include a questionnaire that assesses the memorization of TPE concepts and procedures. Based on the *Revised Bloom’s Taxonomy* [11], there will be a weak alignment or a misalignment of the learning objectives, activities, and assessments (see Fig. 1b).

### Educational outcomes

#### Satisfaction

Educators will complete a questionnaire assessing the extent to which their expectations about the CE activity were met. The questionnaire (©Formaeva, an organization that develops evaluation tools to assess training activities; additional information available at: https://www.formaeva.com/) evaluates the degree to which participants appreciate the activity (1 item, *1 = absolutely no*, *10 = absolutely yes*) and rate its upstream preparation (*n* = 3 items), organization (*n* = 2 items), content (*n* = 4 items), animation (*n* = 3 items), structure (*n* = 2 items), and usefulness (*n* = 2 items), using a 5-point Likert scale (either *1 = strongly disagree*, *5 = strongly agree* or *1 = too short*, 5 = *too long*). Two open-ended questions assess the CE activity strengths and areas of improvement. Formaeva can provide the questionnaire to the reader upon request.

#### Learning

Change from baseline knowledge about TPE will be assessed among educators, who will be asked to complete pre- and post-tests of knowledge about TPE. The questionnaire, comprising eight open-ended questions aligned with the specific objectives of the CE activity (please refer to Additional file 1). Questions were based on current literature on TPE [5, 24]. The questionnaire was pretested among formerly trained educators (*n* = 6) and experienced respiratory educators (*n* = 3) to ensure its face validity. It was also reviewed by experts on TPE (*n* = 1) and CE (*n* = 1), and by the CE activity animators (*n* = 2) for preliminary content validity assessment. The overall score ranges from 0 to 25. A higher score indicates greater knowledge.

#### Self-report of competence

Change from baseline self-report of competence will be assessed in educators using a questionnaire (©Formaeva). This questionnaire measures pre−/post-activity self-report of competence in regard to the following three CE activity objectives: (1) to define TPE, (2) to demonstrate TPE skills while teaching a patient to use and maintain medication delivery devices, and (3) to deliver effective TPE intervention. Again, Formaeva can provide the questionnaire to the reader upon request.

#### Performance

We will measure educators’ post-CE activity performance in delivering high-quality TPE interventions to a real patient by using a published effective TPE rating scale [25, 26]. Twenty operationally defined teaching behaviors assess the following four effective TPE skills: (1) interpersonal skills (*n* = 3 items; e.g. showing respect for patients), (2) presentation skills (*n* = 9 items; e.g. stating learning objectives), (3) essential teaching functions (*n* = 4 items; e.g. providing patients with feedback), and (4) adherence counseling strategies (*n* = 4 items; e.g. negotiating treatment plans) [25]. Each item is scored on a 4-point scale (*0 = absent, 1 = poor, 2 = adequate/good, 3 = excellent use of the skill*). Item scores are averaged to calculate a subscale score (range 0–3, higher scores indicating greater effective TPE skills) [25].

#### Patient outcomes: health status and health resource utilization

After the completion of either CE activity, the newly trained educators will deliver one or several TPE interventions to COPD patients in their professional practice. Among these COPD patients, we will assess change from baseline health status and health resource utilization.

##### Health status

Health status will be measured using the activity and impact components of the COPD-specific version of the *St. George’s Respiratory Questionnaire* (SGRQ-C) [27] and the *COPD Assessment Test* (CAT). Validated French-Canadian versions will be used [28]. Both the SGRQ-C and the CAT are suitable for evaluating COPD patient health status in recent times [27, 29]. The impact dimension of the SGRQ-C will be considered as our primary patient health outcome.

The activity and impact components of the SGRQ-C are comprised of 13 and 20 closed-ended items, respectively [27]. For each component, a weighted score, ranging from 0 (perfect health) to 100 (most severe status), is derived [27]. A difference in SGRQ-C score equivalent to 4 points is considered clinically significant [30]. The SGRQ-C has been developed from the original SGRQ using Rasch analyses [27]. The SGRQ has been previously validated, using correlations with appropriate comparison measures [31].

The CAT is an 8-item instrument that measures health status using 5-point Likert scales [29]. Response options are summed, and the overall CAT score ranges from 0 to 40 (lower scores are better) [29]. The minimal clinically important difference in the CAT score is − 2 [32]. The CAT has been validated using Rasch analyses [33]. Its internal consistency has been shown to be excellent, and its test/re-test reliability has been shown to be good [33]. It has demonstrated responsiveness to change [34].

##### Health education impacts

The eight independent dimensions of the *Health Education Impact Questionnaire* (heiQ), a 40-item questionnaire developed to evaluate proximal TPE outcomes [35], will be measured. These dimensions are: (1) health-directed behaviors, (2) skill and technique acquisition, (3) self-monitoring and insight, (4) positive and active engagement in life, (5) constructive attitudes and approaches, (6) health services navigation, (7) social integration and support, and (8) emotional well-being. Each item of the heiQ uses a 4-point Likert scale (*1 = strongly disagree, 4 = strongly agree*, except for the eighth dimension, which uses a reversed scale) [35]. Response options are averaged, and higher scores are better [35]. The heiQ development was based on a *Program Logic Model*, *Concept Mapping*, and interviews with stakeholders [35]. Item response theory and structural equation modeling were used to assess its psychometric properties [35]. The heiQ was found to have high construct validity and to be reliable [35]. In this study, we will use the Canadian-French version of the heiQ [36].

##### Health resource utilization

The number of unscheduled doctor visits, emergency room visits, and hospitalizations in the six preceding months will be measured using three items derived from the Survey on Living with Chronic Diseases in Canada (SLCDC) [37] and available at: http://www23.statcan.gc.ca/imdb-bmdi/instrument/5160_Q6_V1-eng.pdf. To ensure the face validity of the SLCDC, questions were framed in collaboration with respiratory experts and qualitatively tested using face-to-face interviews [37].

### Confounding, professional, clinical, and sociodemographic variables

Determinants of educational outcomes identified a priori will be measured (see Table 3). To characterize study participants, we will collect data on attendees’ jobs, undergraduate studies, the number of years of professional practice, and former participation in CE activities, using a standardized form. In COPD patients, we will measure disease severity that is classified by symptoms and disability [38], along with marital status, highest attained level of education, and annual family income, using items derived from the *Quebec Survey on Cardiovascular Health* (QSCH) [39].

**Table 3:** List of possible confounding factors

Educational outcome	Confounding variable	Units, categories, or range	Instrument
Educators’ satisfaction Educators’ learning Educators’ competence Educators’ performance	Age [12]	in years	Standardized form
	Level of education [12]	<University	Standardized form
		≥University	
	Motivation to participate in the CE activity [12]	Score: 0–6	Adapted from the MSLQ [58]
COPD patients’ outcomes	Smoking history [59]	in packs-year	SLCDC [37]
	Dyspnea [60]	*Medical Research Council dyspnea scale*, grade: 1–5	SLCDC [37]
	Social support [59]	Yes	SLCDC [37]
		No	
	Comorbidity [60]	Yes	SLCDC [37]
		No	
	Respiratory tract infections [59]	Yes	SLCDC [37]
		No	
	Body mass index [60]	< 21	QSCH [39]
		≥21 kg/m^2^	
	Age [61],	in years	QSCH [39]
	Gender [62]	Women	QSCH [39]
		Men	
	Previous exacerbations in the six preceding months [59, 60]	Yes	Telephone interviewer-administered questionnaire [63]
		No	
	Levels of anxiety [60, 64]	Score: 0–21	HADS [65]
	Levels of depression [60, 64]	Score: 0–21	HADS [65]

Depending on the educational outcome, these variables will be measured in educators or in COPD patients

*HADS* Hospital Anxiety and Depression Scale, *MSLQ* Motivated Strategies for Learning Questionnaire, *QSCH* Quebec Survey on Cardiovascular Health, *SLCDC* Survey on Living with Chronic Diseases in Canada

### Participant timeline

**Fig. 2:**
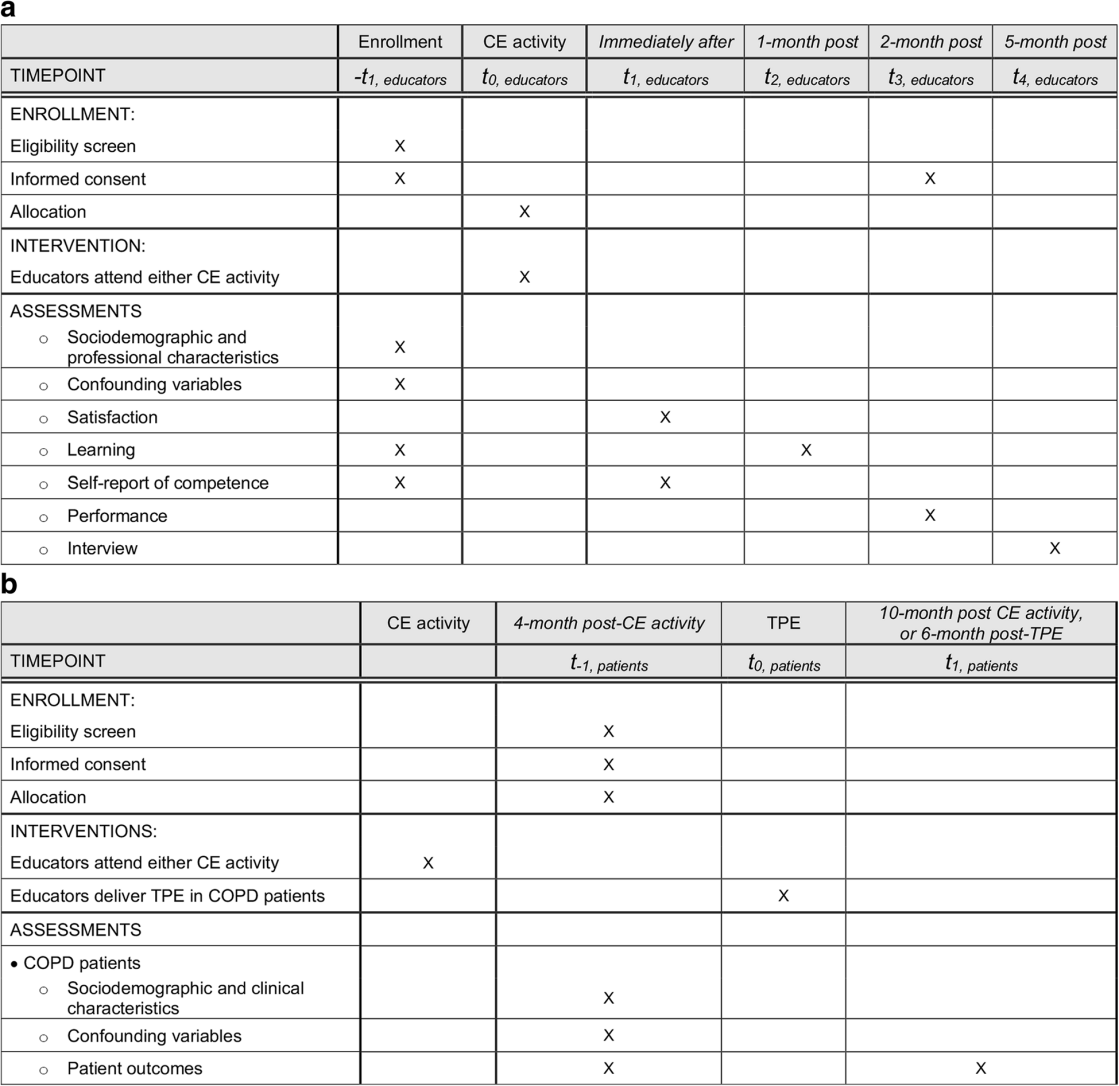
SPIRIT flow diagram: Educators’ and patients’ timelines for the schedule of enrolment, interventions, and assessments Derived from the SPIRIT statement [57]. **a**. Educator timeline Educators will be enrolled and will be asked to complete baseline measurements on the morning of the CE activity (*t*_*-1, educators*_). Post-CE activity measurements will be undertaken immediately after the CE activity (*t*_*1, educators*_), for satisfaction and self-report of competence, at 1-month post-activity (*t*_*2, educators*_), for learning, and at 2-month post-activity (*t*_*3, educators*_), for performance. Interviews will be conducted among educators 5 months after the CE activity (*t*_*4, educators*_). **b**. Patient timeline. Four months after attending either CE activity, educators will perform TPE in COPD patients. Patient outcomes will be measured prior to TPE, at (*t*_*-1, patients*_). Six months later, at (*t*_*1, patients*_), post-TPE measurements will be undertaken in COPD patients

Figure 2 illustrates the schedule for enrollment, interventions, and assessments. The measurement time points have been chosen in accordance with the *Expanded Outcomes Framework for Planning and Assessing Continuing Medical Education Activities* [15] (e.g. satisfaction and self-report of competence are measured immediately after a CE activity) and feasibility issues (e.g. time for institutional feasibility approval for performance and patient outcome assessments). As illustrated in Fig. 2a, educators will be enrolled and will be asked to complete baseline measurements on the morning of the CE activity (*t*_*-1, educators*_). Among educators, post-activity measurements will be undertaken immediately after the CE activity (*t*_*1, educators*_), for satisfaction and self-report of competence, at 1-month post-activity (*t*_*2, educators*_), for learning, and at 2-month post-activity (*t*_*3, educators*_), for performance. Interviews will be conducted among educators at (*t*_*4*_*,* educators), after quantitative measurements (details on the interviews will be given in section “Interviews with educators” below).

Four months after the CE activity, newly trained educators will deliver TPE to COPD patients. In COPD patients, measurements will be undertaken prior to TPE, at (*t*_*-1, patients*_), and six months after TPE, at (*t*_*1, patients*_).

### Sample sizes

The size of our sample of educators was calculated on the basis of our fourth study hypothesis, that predicted that post-CE activity performance levels will be higher in the experimental group compared to the other [13, 14]. In a previous study, educators were found to have a score of 0.89 on the effective TPE rating scale [25]. We think that the educators of the comparison group will also have this score. We expect the educators of the experimental group to have a score of 2.00, that is considered as good on this scale [25]. Therefore, we calculated that a sample size of seven educators per group was required to detect a group difference of 1.11 point (standard deviation, or SD = 0.63; type II error = 0.20, or 80% power; type I error = 0.05; two-sided test).

The size of our sample of COPD patients was calculated on the basis of our fifth study hypothesis, that predicted that patient outcomes will be further enhanced in COPD individuals who will meet the newly trained COPD educators of the experimental group, compared to the other [13, 14]. We expect the COPD patients, who will meet the newly trained COPD educators of the comparison group, to experience a change at 6-month follow-up equivalent to − 2 points on the impact dimension of the SGRQ-C, similar to the patients who were allocated to a previous usual care intervention [40]. Consistent with previous results [40], we expect the COPD patients, who will meet the newly trained COPD educators of the experimental group, to make an improvement equivalent to + 9 points. Using the SAS generalized estimating equation macro for controlled clinical trials with repeated measurements on the same individuals [41], we estimated that a sample size of 94 patients per group was required to detect a between-group difference of 11 points (SD = 19; type II error = 0.20, or 80% power; type I error = 0.05; two-sided test).

To account for possible losses to follow-up, to ensure Gaussian distributions, and because it was considered as feasible to recruit ≈5 COPD patients per educator, we hope to recruit 25 educators per group and 125 COPD patients per group (≈5 patients per educator). The CE activities will be delivered on several occasions from June 2016, and thereafter, to achieve the required sample sizes of both educators and patients.

### Assignment of interventions

The CE activities will be organized on behalf of the *Quebec Respiratory Health Education Network* twice per year, either in Quebec City or Montreal, Quebec, Canada. Upon request, the *Quebec Respiratory Health Education Network* may decide to schedule additional CE activities outside these two cities. Educators, who will participate in the CE activities held from June 2016 to October 2017, will be allocated the comparison group, whereas those attending the activity after November 2017 will be assigned to the experimental group.

### Blinding

Data analysts will be blinded to interventions, along with study participants, who will not be aware that there will be two different CE activities.

### Recruitment

Before either CE activity, the study coordinator (M.G.) will invite all educators to participate in the study and will be in charge of their recruitment. Four months after the CE activity, the newly trained educators will invite their COPD patients to participate in the study. Educators will communicate the names and contact information of the COPD patients who will accept the invitation to take part in the study to the study coordinator. Then, trained research assistants will be responsible for the recruitment of COPD patients. We plan to stop the recruitment when we will reach the targeted sample sizes of both educators and COPD patients.

### Data collection methods

Questionnaires measuring educators’ satisfaction, learning (baseline/post-activity knowledge), competence, and confounding factors will be self-administered. For satisfaction, baseline knowledge, pre−/post-activity self-report of competence, and confounding factors, data will be collected on the CE activity site. Data on post-activity knowledge will be collected on the educators’ work site. We will send reminders to educators who will not return the post-activity knowledge questionnaire.

To measure performance for each educator, we will videotape one TPE intervention delivered to a real COPD patient in each educator’s professional practice. The study coordinator will send the camera to the educator by mail. Upon reception of the camera, the educator will invite the first patient to whom he or she will deliver TPE to participate in the study. If the patient refuses, then the educator will ask the following patient, and so on. We will ask educators to place a GoPro HERO Session™ camera (GoPro, San Mateo, CA, USA) on their desk. The camera has the height of 38 mm, the width of 38 mm, and the depth of 36.4 mm. It starts recording when pushing a single button and captures up to a 2-h ultra-wide field of view video in .mp4 file format.

Data on COPD patient health status, resource utilization, and confounding factors will be collected via telephone interviews with trained research assistants. The SLCDC is an interviewer-administered instrument. Previous studies have shown that the SGRQ and the CAT produce similar results when interviewer-administered [42, 43]. The heiQ, along with the *Hospital Anxiety and Depression Scale* (HADS), used to measure two confounding factors, will be mailed to patients and self-administered at each of the patients’ homes. Both the heiQ and the HADS could be interviewer-administered, upon request, to avoid missing data.

### Data management

We will perform independent double data entry for numeric data (satisfaction, self-report of competence, patient outcomes, confounding factors). Two individuals will independently rate attendees’ learning and performance. When attributing knowledge scores, independent raters will be blinded to the measurement time point. They will score videotapes in random order. We will calculate interrater reliability using an intra-class correlation coefficient [44, 45]. Consensus will resolve disagreements. If necessary, a third reviewer will be consulted. All data will be stored on a secure server to prevent unauthorized access and loss of participant data.

### Statistical analyses

Data will be analyzed by intention-to-treat [46]. The experimental group will be compared to the comparison group for all statistical analyses. To compare participant characteristics, we will use χ^2^ test for binary outcomes and *t*-test for continuous outcomes. In educators, we will compare post-CE activity satisfaction and performance scores using multivariate linear regression models [47]. We will compare, in educators, change from baseline knowledge scores and change from baseline self-report of competence scores, and, in COPD patients, change from baseline patient outcome scores or counts, using generalized linear or Poisson mixed models with appropriate interaction terms (group × time) [48]. We will assume a specified form of covariance structure among the two repeated measurements (knowledge: *t*_*2, educators*_ versus *t*_*-1, educators*_; competence: *t*_*1, educators*_ versus *t*_*-1, educators*_; COPD patient outcomes: *t*_*1, patients*_ versus *t*_*-1, patients*_). We will assume a specified form of covariance structure among the two repeated measurements (knowledge: *t*_*2, educators*_ versus *t*_*-1, educators*_; competence: *t*_*1, educators*_ versus *t*_*-1, educators*_; COPD patient outcomes: *t*_*1, patients*_ versus *t*_*-1, patients*_). Estimates and standard errors will be based on a restricted likelihood function given the observed data. Specifying an unstructured covariance matrix, we will handle possible missing values at follow-up [49]. Depending on the variable type, we will calculate differences in means and counts with corresponding 95% confidence intervals [47, 48]. Model assumptions will be assessed. Determinants of educational outcomes identified a priori will be included in statistical models if they result in a > 10% change in the differences in means and counts [50]. We will examine the residuals to assess goodness-of-fit [47, 48]. We also plan to perform independent variable transformations if model assumptions are not met [51] or to use ordinal or binomial regression models. We will use an up-to-date version of SAS (Cary, NC, USA) to conduct all statistical analyses and two-sided *p*-values with α ≤0.05 level of significance for all tests.

### Interviews with educators

We will conduct telephone interviews with educators of both the experimental and comparison groups to further explain our quantitative results [21]. We will purposefully select educators for interviews, based, for instance, on their performance scores or their professional characteristics. Based on the levels of assessment of the *Expanded Outcomes Framework for Planning and Assessing Continuing Medical Education Activities* [15], we will develop a semi-structured interview guide. The guide will also question the educators about the organizational support that they receive in their work setting in regard to TPE intervention delivery.

### Qualitative analyses

We will audiotape the interviews, transcribe interviews verbatim, and randomly check selected extracts of transcripts to ensure that there are no mistakes [21]. We will import data to QSR NVivo 11 software® and conduct content analysis [52]. Two members of our team will independently read and code three interviews’ transcripts, using an inductive approach [52]. They will compare their coding and debrief in order to develop the first version of the codebook. Based on this first version, they will independently assign codes to the data of the other interviews. We will compare their codes, resolve disagreements by consensus, and refine the codebook. Codes will be sorted into categories and the categories into themes. We will conduct interviews until saturation is reached [21]. Findings from the final analysis will be presented to educators to determine their accuracy [21].

### Integration

In the discussion section of the published articles, results will be integrated in order that qualitative findings help expand or explain the quantitative ones [21].

### Dissemination

We plan to communicate our results to the *Quebec Respiratory Health Education Network*, which designed and delivers the CE activities. We will also communicate them to study participants, Quebec Ministry of Health, and researchers, via publications and presentations in local, provincial, national, and international meetings. We intend to have no publication restriction.

## Discussion

Based on the framework by Moore et al. [15], our study will aim to compare the impacts of two CE activities on TPE that target COPD educators in regard to five educational outcomes.

### Strengths

Previous pre/post studies [53–55] have evaluated the impact of CE activities targeted at COPD educators on some, but not all educational outcomes, as defined by Moore et al. [15]. None of these previous studies evaluated educators’ organizational support in regard to TPE intervention delivery, even though the organizational support is a major factor to consider when assessing the impact of a CE activity [12]. To the best of our knowledge, this study is the first controlled mixed methods study to compare the impact of two CE activities on TPE in regard to five educational outcomes, while qualitatively documenting how educators are supported by their organization.

In contrast to previous studies [53–55], our study will objectively assess educators’ learning, using pre- and post-tests of knowledge, as suggested by Moore et al. [15]. Our study will also evaluate educators’ performance levels in delivering high-quality TPE interventions, based on observation measures of performance in each of the educator’s professional practice.

### Limitations

We expect our study to have limitations. First, non-differential measurement errors might occur, because:

1. we designed an ad hoc assessment test to measure educators’ learning, due to the fact that we wanted the knowledge questionnaire to be aligned with the learning objectives of both CE activities;
2. we will assess educators’ performance levels in delivering high-quality TPE education to a single real patient, instead of videotaping educator encounters with both real and standardized patients, as it has been suggested [56];
3. educators’ performance will only be measured after the CE activity;
4. we will only perform a subjective assessment of changes in patient outcomes, due to organizational constraints.

Second, because the study is a pragmatic one, we expect that there could be some heterogeneity between the TPE interventions that will be delivered by each of the educators’ professional practice. Because what is referred to TPE may differ from a setting to another, heterogeneity is likely to have an impact on changes from baseline patient outcomes. Nevertheless, interviews with attendees will help to describe the implementation of TPE programs better, and, in turn, to explain our study results better.

Finally, we will compare, in the present study, two CE activities that will include two independent components: (1) the number of attendees (< 10 versus ≥ 10) and (2) the learning format (active versus passive). In contrast to a factorial design, our study design will not allow us to understand the effect of each independent component upon the educational outcomes.

### Perspectives

To the best of our knowledge, our study is the first controlled mixed methods study to compare the impact of two CE activities on TPE in regard to five educational outcomes. The experimental CE activity was designed to promote the achievement of higher-order cognitive processes and align its learning objectives, activities, and assessments. We believe this study will serve as a model for evaluating CE activities on TPE. Results from this study could increase COPD educators’ performance levels in delivering effective TPE interventions, and, in turn, COPD patient outcomes.

## Additional file

Additional file 1: Questionnaire on attendees’ learning. The questionnaire on attendees’ learning comprises eight open-ended questions aligned with the CE activity specific objectives. A professional translator translated the items from French to English. (DOCX 46 kb)

## Abbreviations

CAT: COPD Assessment test
CE: Continuing education
CI: Confidence interval
COPD: Chronic obstructive pulmonary disease
HADS: Hospital anxiety and depression scale
heiQ: Health education impact questionnaire
MSLQ: Motivated strategies for learning questionnaire
QSCH: Quebec survey on cardiovascular health
SD: Standard deviation
SGRQ-C: COPD-specific version of the St. George’s respiratory questionnaire
SLCDC: Survey on living with chronic diseases in Canada
TPE: Therapeutic patient education
