# Supplementary material for: Comparative impact of two continuing education activities targeted at COPD educators on educational outcomes: protocol for a non-randomized controlled study using mixed methods
Source: BMC Health Serv Res. 2018 Jun 18;18:460. doi: 10.1186/s12913-018-3284-6 (PMC6006567; doi:10.1186/s12913-018-3284-6)
Supplement: Supplementary file 1 — Questionnaire on attendees’ learning. The questionnaire on attendees’ learning comprises eight open-ended questions aligned with the CE activity specific objectives. A professional translator translated the items from French to English. (DOCX 46 kb) [file 12913_2018_3284_MOESM1_ESM.docx]

1. In your opinion, what is the difference between educating, teaching and informing?

|  |
| --- |
|  |
|  |
|  |
|  |

1. Imagine Mr. Johnson, a patient with COPD, says to you:

| Mr. Johnson: | I know I have to use my green and grey inhaler [Spiriva®] every day and I really want to use it to improve my breathing, but I have to admit that I often forget ... |
| --- | --- |
| Based on what you think is an appropriate educational approach, how would you go about helping Mr. Johnson use his medication properly, that is, once a day, every day, as prescribed? | |
|  | |
|  | |
|  | |
|  | |

1. Imagine that Mr. Johnson, a patient with COPD, says to you:

| Mr. Johnson: | Mr. Johnson I don’t really remember how to use my green and grey inhaler [Spiriva®] anymore ... |
| --- | --- |
| Based on what you think is an appropriate educational approach, how would you go about helping Mr. Johnson learn the correct way to take his medication? | |
|  | |
|  | |
|  | |
|  | |

1. Imagine Mr. Johnson, a patient with COPD, says to you:

| Mr. Johnson: | I don’t want to stop smoking. It’s the only thing left that I enjoy and my mother, who died when she was 90, smoked all her life and never had to use an inhaler. |
| --- | --- |
| Based on what you think is an appropriate educational approach, how would you go about helping Mr. Johnson stop smoking? | |
|  | |
|  | |
|  | |
|  | |

1. Here is part of an exchange between an educator and Mr. Johnson, a patient with COPD:

| Educator: |  | I see in your record that Dr. Tremblay renewed your prescription for Advair®. |
| --- | --- | --- |
| *Mr. Johnson frowns.* | | |
| Educator: |  | Your purple inhaler ... |
| *Mr. Johnson’s face clears and he takes an inhaler device out of his bag.* | | |
| Mr. Johnson: |  | Ah, you mean this inhaler? |
| Educator: |  | Exactly.  We talked about this medication the last time we met. Do you need me to explain what it is for again today? |

- 1. Why do you think the educator mentioned the colour of the inhaler device?

|  |
| --- |
|  |

- 1. Based on what you think is an appropriate educational approach, how else could the educator have found out if Mr. Johnson knew what his medication was for?

|  |
| --- |
|  |

1. Imagine that you are meeting with Mr. Johnson, a patient with COPD, for a follow-up educational intervention. Based on what you think is an appropriate educational approach, how would you go about assessing whether Mr. Johnson follows his action plan correctly when there are signs that his symptoms are worsening?

|  |
| --- |
|  |
|  |
|  |
|  |

Questions 7 and 8 are about the following excerpt from an exchange between an educator and Mr. Johnson, a patient with COPD.

1. What is your assessment of the quality of the educator’s educational intervention? If you agree with what the educator says, put a (+) in the “Assessment” column. If you do not agree with what the educator says, put a (-) in the “Assessment” column. Give a rationale for each (+) or (-) element based on what you think is a quality educational intervention.
2. How could you improve the interaction between the educator and the patient? For each (-) element, suggest an alternative reply for the educator.

|  | Question 7:  Assessment (+/–) and rationale | Question 8:  Suggested alternative |
| --- | --- | --- |
| Mr. Johnson:  I find it hard to explain what COPD is to my family and friends... |  |  |
| Educator:  Actually, you can say that COPD is characterized by progressive and partially reversible airway obstruction, hyperinflation of the lungs, systemic manifestations and increasingly frequent and severe exacerbations. |  |  |
